# Supplementary material for: Trypsin is a coordinate regulator of N and P nutrients in marine phytoplankton
Source: Nat Commun. 2022 Jul 12;13:4022. doi: 10.1038/s41467-022-31802-6 (PMC9276738; doi:10.1038/s41467-022-31802-6)
Supplement: Supplementary file 1 — Supplementary Information [file 41467_2022_31802_MOESM1_ESM.pdf]

Trypsin is a coordinate regulator of N and P nutrients in marine phytoplankton

Yanchun You[1], Xueqiong Sun[1], Minglei Ma[1], Jiamin He[1], Ling Li[1], Felipe Wendt Porto[2], Senjie Lin[1,2*]

Affiliations:

[1]State Key Laboratory of Marine Environmental Science, College of Ocean and Earth Sciences, Xiamen University, Xiamen, China

[2]Department of Marine Sciences, University of Connecticut, Groton, CT, United States

*To whom correspondence should be addressed: S.L. (senjie.lin@uconn.edu)

Supplementary Data

Supplementary Table 1 Gene characteristics of trypsin in *P. tricornutum*.

Name	Gene ID	pI/MW (kDa)	CDS length (bp)	Chr.	Genomic location
PtTryp1	Phatr3_J3211	8.89/67.68	1848	1	478577-479386
PtTryp2	Phatr3_J54319	4.34/66.26	1854	5	598808-599891
PtTryp3	Phatr3_J45961	5.05/40.78	1101	8	783487-784064
PtTryp4	Phatr3_J13240	7.68/49.60	1401	11	681318-682858
PtTryp5	Phatr3_J37254	4.57/39.66	1107	12	525221-526219
PtTryp6	Phatr3_J49223	9.1/49.81	1392	21	271661-272994
PtTryp7	Phatr3_J49602	4.53/59.37	1677	23	167242-169127
PtTryp8	Phatr3_J7679	3.98/76.63	2181	23	460956-461270
PtTryp9	Phatr3_J40462	4.6/63.87	1764	23	467436-468962
PtTryp10	Phatr3_J49772	6.21/61.61	1749	24	251021-252769

Supplementary Table 2 Subcellular localization prediction of trypsin in *P. tricornutum*.

	NucPred	TMHMM	TargetP	Euk-mPLoc 2.0	Plant-mPLoc	SignalP 3.0	SignalP 4.0	MitoFates	HECTAR v1.3
PtTryp1	-	-	-	Cytoplasm	Chloroplast	-	-	-	-
PtTryp2	-	-	S	Extracell	Chloroplast	+	+	-	-
PtTryp3	-	1 (297-316)	S	Extracell	Nucleus	+	+	-	-
PtTryp4	-	-	-	Extracell	Cell wall Nucleus	-	-	-	-
PtTryp5	-	-	C	Peroxisome	Chloroplas	+	+	-	-
PtTryp6	-	-	M	Extracell	Cell wall Golgi apparatus	-	-	-	-
PtTryp7	-	-	C	Extracell	Cell wall	-	-	-	-
PtTryp8	-	1 (535-557)	S	Extracell	Cell membrane Cell wall Chloroplast	+	+	-	-
PtTryp9	-	-	C	Extracell Nucleus	Nucleus	+	+	-	-
PtTryp10	-	-	M	Nucleus	Chloroplast	+	+	-	-

Supplementary Table 3 Information of 11 classic housekeeping gene in *P. tricornutum.*

Gene name	Gene ID
Actin16	Phatr3_J29812
Actin12	Phatr3_J29136
TubA (tubulin α chain)	Phatr3_J54534
TubB (tubulin β chain)	Phatr3_J21122
GAPDH4 (glyceraldehyde-3-phosphate dehydrogenase)	Phatr3_J51128
TBP (TATA box binding protein)	Phatr3_J10199
EF1 a (translation elongation factor a subunit)	Phatr3_J18475
Ub (Ubiquitin)	Phatr3_J29166
Histone H4	Phatr3_J34971
RPS (ribosomal protein small subunit 30S)	Phatr3_J10847
CdkA	Phatr3_J20262

Supplementary Table 4 Predicted transcription factors co-regulated with *PtTryp2*.

Gene ID	TF class
Phatr3_EG01965	HSF
Phatr3_J14805	E2F/DP
Phatr3_J35419	HSF
Phatr3_J44570	HSF
Phatr3_J45392	HSF
Phatr3_J48955	CPP
Phatr3_J51933	bZIP
Phatr3_J50624	C3H
Phatr3_J48179	MYB
Phatr3_EG02570	MYB_related

Supplementary Table 5 Information of primers used in this study.

Primer name	Sequence 5'-3’	Length (bp)	Product Length (bp)	Note
PtTryp1-F	TATTTTCGTCTGATGGCTTTGTGC	24	87	qRT-PCR
PtTryp1-R	TGCGACCGTCCGTCAAAGTAA	21		
PtTryp2-F	GGTGGAGAAGATGCCAGTGTAGG	23	105	qRT-PCR
PtTryp2-R	GCAGTGGGCAGCAGTGAGAAC	21		
PtTryp3-F	TGTTCCTTATGAGGGCCAGTATGTC	25	88	qRT-PCR
PtTryp3-R	CTCCTGTAGGGTGTCGGCTTTAGA	24		
PtTryp4-F	GCGATGGCTATTCTACCGTTCAC	23	137	qRT-PCR
PtTryp4-R	CACAGCGAAGTCATAGCGAAACA	23		
PtTryp5-F	TATCTCCACCGATCCGCAACT	21	107	qRT-PCR
PtTryp5-R	CGTATTCACGCCGACTACTTCC	22		
PtTryp6-F	CTGTCAGGGAGATTCTGGTGGTC	23	87	qRT-PCR
PtTryp6-R	CTATCGCCACAACCGACACC	20		
PtTryp7-F	TGATCCTCGCTGACCCAGTGA	21	116	qRT-PCR
PtTryp7-R	ACCGTAAAGGGTACTGCCAAAGC	23		
PtTryp8-F	TGACAACCTGACGGTAATGGGC	22	94	qRT-PCR
PtTryp8-R	GAAATCGACGGCGAGTAGGGT	21		
PtTryp9-F	CGGTCAGCACCAGCCTCTTTC	21	116	qRT-PCR
PtTryp9-R	GGACGCATCCTCCTCCGCTA	20		
PtTryp10-F	CACACTGACGGCAGGCATTATTT	23	94	qRT-PCR
PtTryp10-R	GGCGGCGTCGGTCTGAATA	19		

PtTBP-F	ATTCCGCCTTTGCCAGTTACGA	22	111	qRT-PCR
PtTBP-R	CAGTAATGACTATCTTTCCGCTCACGA	27		
PtRPS-F	CGAAGTCAACCAGGAAACCAA	21	166	qRT-PCR
PtRPS-R	GTGCAAGAGACCGGACATACC	21		
PtHPRT-F	GATCTGGCGGCTATCATAAAAGAGG	25	130	qRT-PCR
PtHPRT-R	TGTCATATCCTTGGCACAGGTCTTG	25		
PtTryp2-gRNA-F	TCGAGGTGGAGAAGATGCCAGTGT	24		Adapter for *PtTryp2* knockout
PtTryp2-gRNA-R	AAACACACTGGCATCTTCTCCACC	24		Adapter for *PtTryp2* knockout
PtTryp2-KO-F	GCAAAGAGAACCTATTCGGAACTAAG	26	637	Screening for *PtTryp2* knockout mutants
PtTryp2-KO-R	CTTTTTGGAGAATGTTACTGCCAGA	25		
diaCas9-F	TTGGAAACATTGTGGACGAGGT	23	464	Screening for *PtTryp2* knockout mutants
diaCas9-R	AGGAGGGTCAGGTCTTGGTGAT	23		
eGFP-F	GCTCTAGAATGGTGAGCAAGGGCGAGGA	28	717	*eGFP* overexpression
eGFP-R	CCCAAGCTTTTAGTGGTGGTGGTGGTGGTGCTTGTACAGCTCGTCCATGCCG	52		
PtTryp2-OE-F	CGGAATTCATGAAGTTTTTACCAACGGCTCTAGC	34	852	*PtTryp2* overexpression
PtTryp2-OE-R	CGGGATCCCGCACAGACTTGTCCATTGATCC	31		

Supplementary Table 6 Stability evaluation of reference genes expression.

Method	Ranking Order (Better--Good--Average)		
	1	2	3
Delta CT	*PtTBP*	*PtHRPT*	*PtRPS*
BestKeeper	*PtTBP*	*PtRPS*	*PtHRPT*
Normfinder	*PtTBP*	*PtHRPT*	*PtRPS*
Genorm	*PtTBP* | *PtHRPT*		*PtRPS*
Recommended comprehensive ranking	*PtTBP*	*PtHRPT*	*PtRPS*

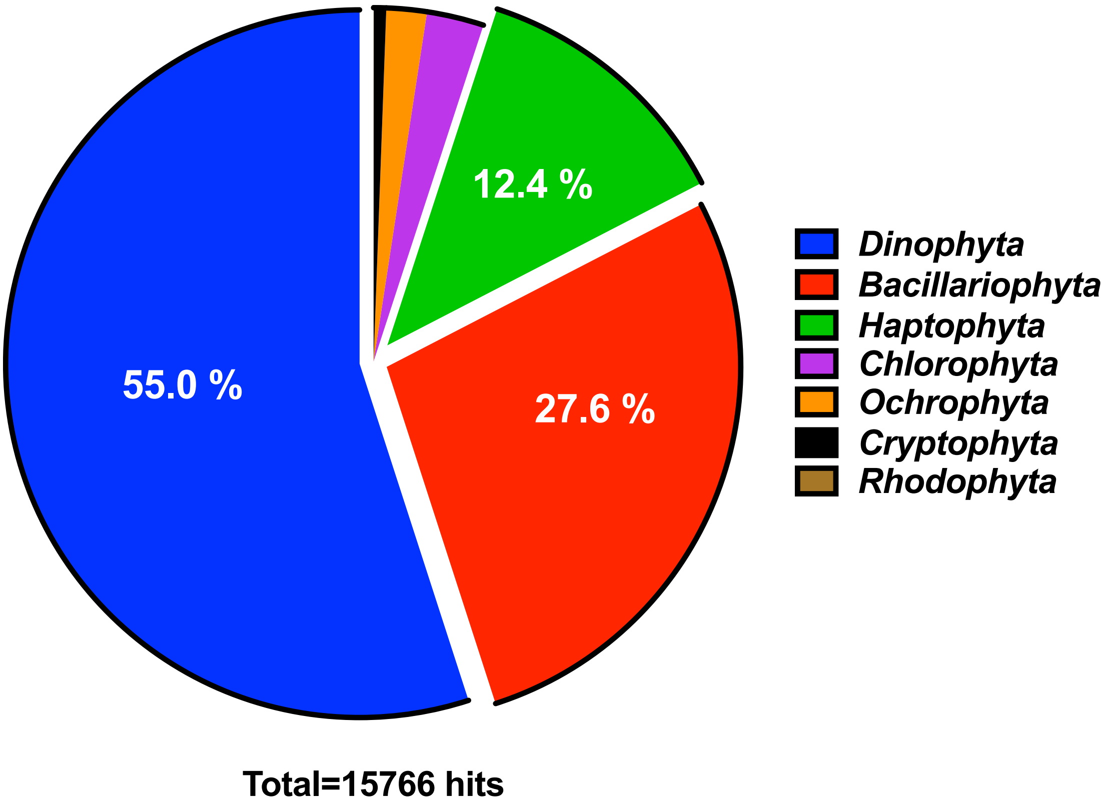

Supplementary Figure 1 Taxonomic distribution of identified phytoplankton trypsin homologs from *Tara* Oceans Unigenes. Datasets were obtained from the Marine Atlas of *Tara* Oceans Unigenes (MATOU) database (https://tara-oceans.mio.osupytheas.fr/ocean-gene-atlas/). Source data are provided as a Source Data file.

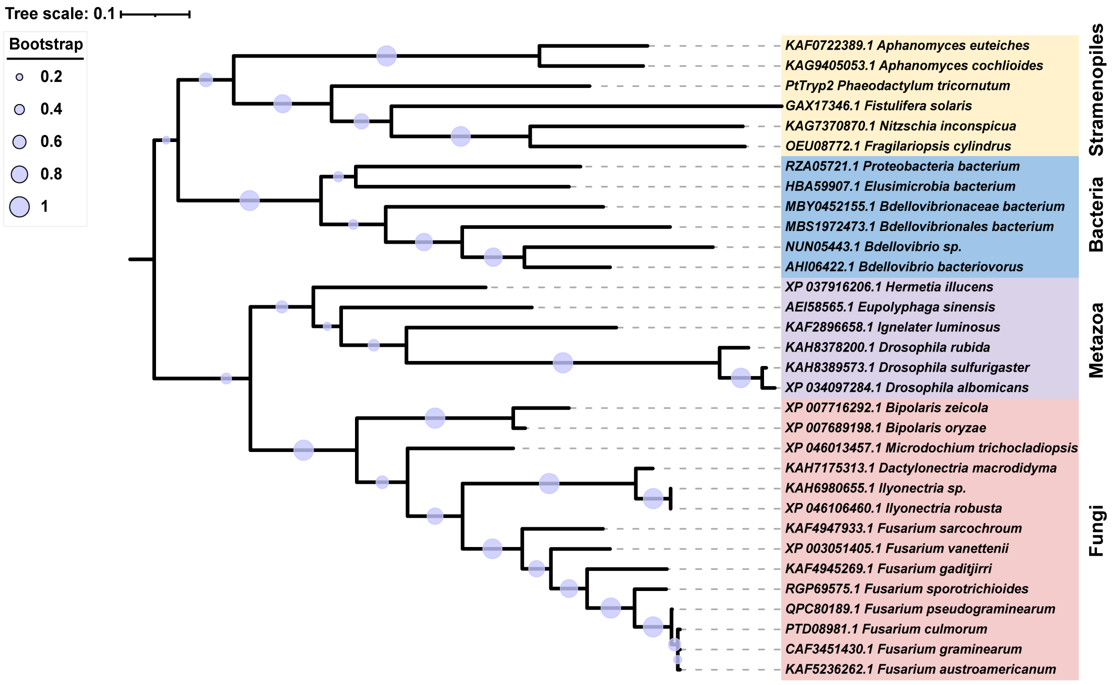

Supplementary Figure 2 Phylogenetic relationships of *PtTryp2* with different trypsin protein sequences. The Maximum Likelihood phylogenetic tree was constructed using MEGA X software with bootstrap test of 1000 times, based on a discrete Gamma distribution of evolutionary rate variations, which was recommended by the results of Poisson correction model. The resulting tree file was visualized with iTol (https://itol.embl.de). The tree shows that different class of trypsins are distinct, and the *P. tricornutum* trypsin is clustered with most of the diatom trypsins, which share a closer relation with the trypsins from bacteria than metazoans and fungi. Scale bar indicates amino-acid substitutions per site. Source data are provided as a Source Data file.

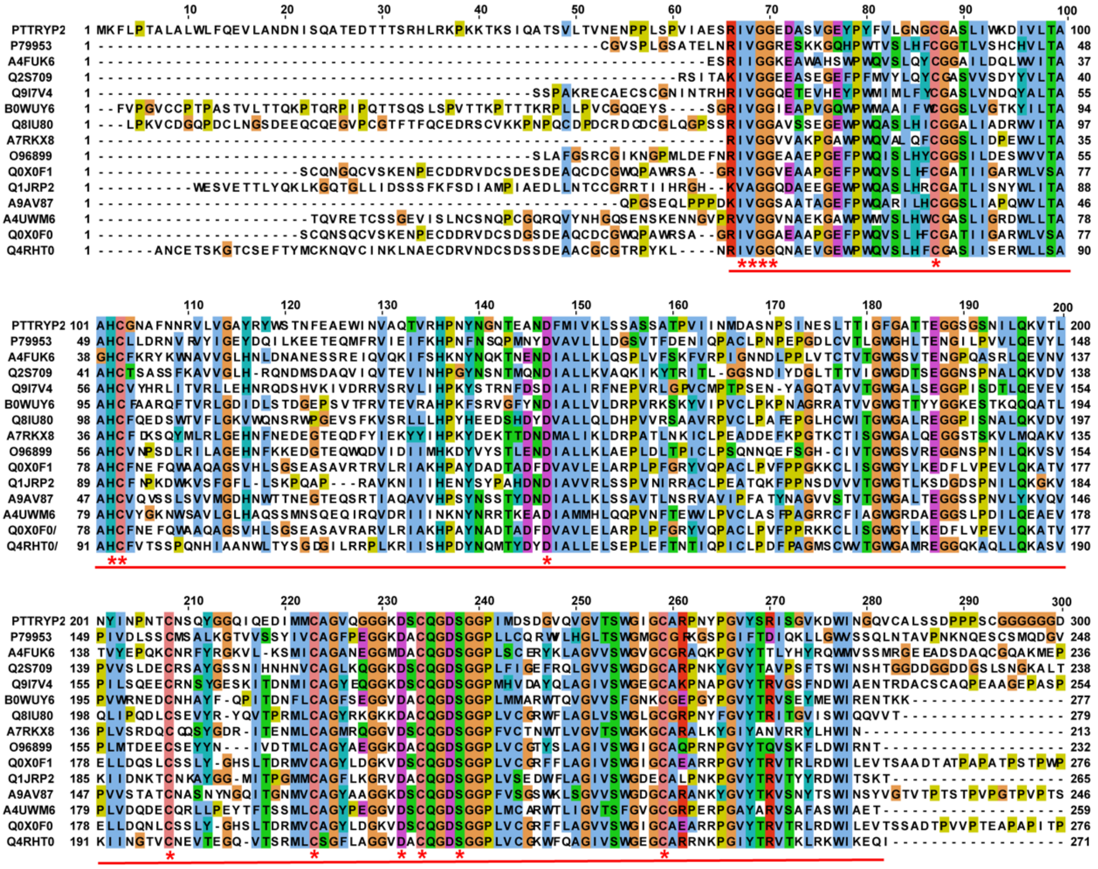

Supplementary Figure 3 Alignment of trypsin amino acid sequences. The important residues of trypsin domain are conserved in *PtTryp2*. The *PtTryp2* trypsin sequences, along with the other trypsin sequences downloaded from EBI database, were aligned using CLUSTAL W. Dashes represent gaps introduced for the alignment. Residues that are identical are represented by same background colors. The trypsin domain and the structurally important residues (Rypniewski et al. 1994) are indicated by red line and red asterisks, respectively. These include the trypsin catalytic triad (Ser-His-Asp), the substrate binding site, the Cys residues at the conserved disulphide bridges, and the amino terminus of the mature trypsins (peptide IVGG). Source data are provided as a Source Data file.

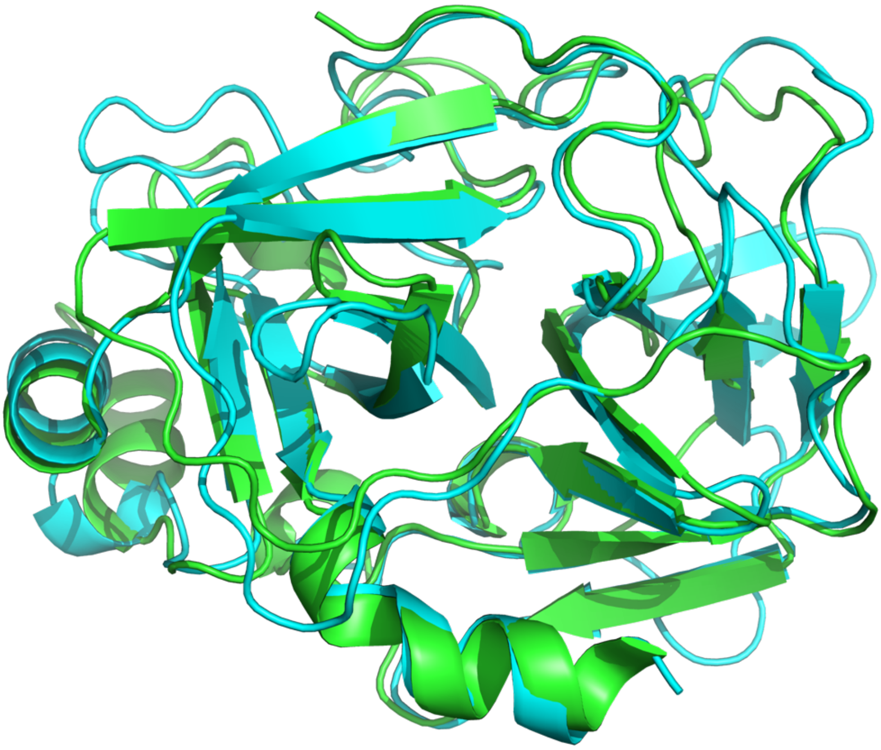

Supplementary Figure 4 Protein structure alignment and superposition of *PtTryp2* against BtTryp. It indicates that *PtTryp2* and animal trypsin have a highly similar architecture. The structure of *PtTryp2* was predicted by Phyre2. The structure of BtTryp (*Bos taurus*) was download from PDB database (PDB id: 4BNR). The structures of *PtTryp2* and BtTryp are colored green and cyan, respectively.

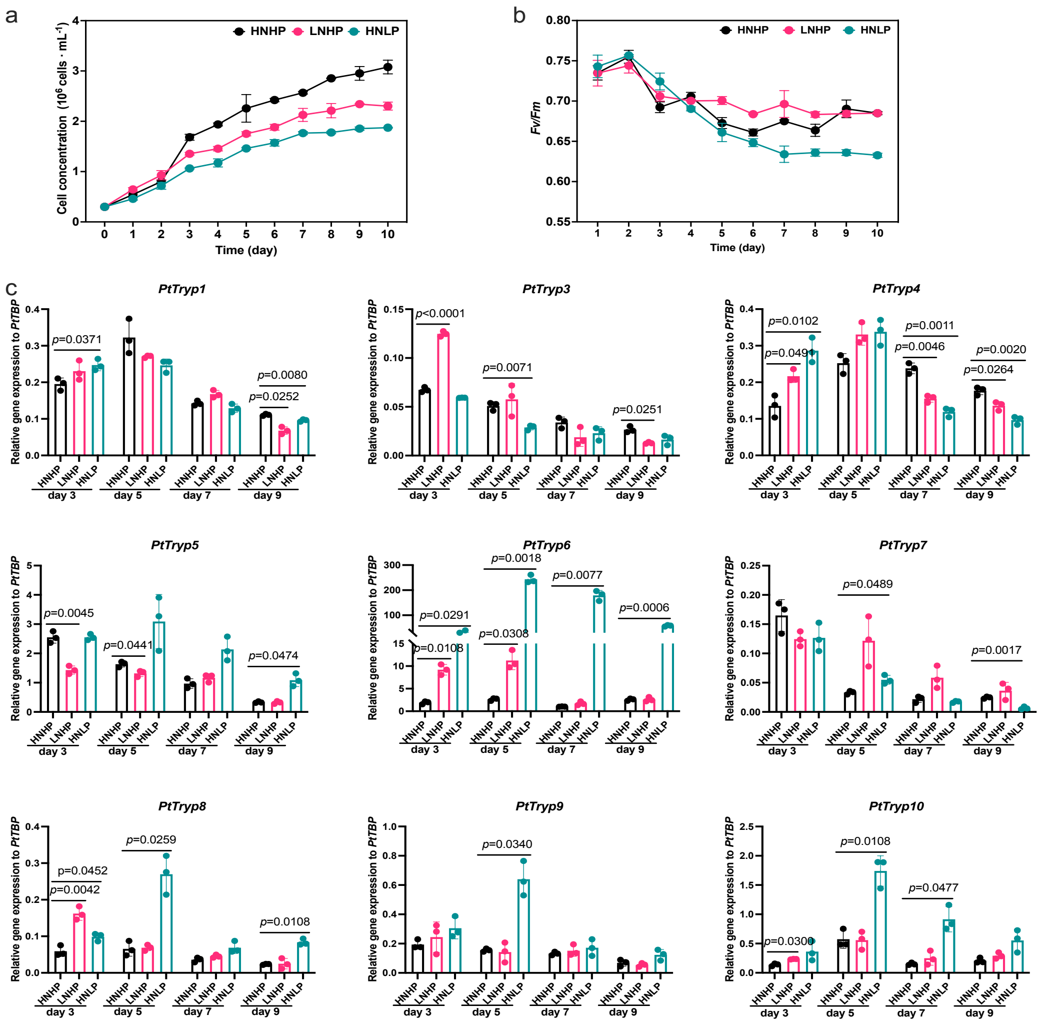

Supplementary Figure 5 Physiological parameters and expression patterns of identified trypsin genes in *P. tricornutum* under different conditions. The WT cells grown under nutrient-replete (HNHP), N-depleted (LNHP), and P-depleted (HNLP) conditions. **a**, Cell concentration. Data are presented as mean values ± SD (n=3 biologically independent samples). **b**, Photosynthetic efficiency as indicated by *Fv/Fm*. Data are presented as mean values ± SD (n=3 biologically independent samples). **c**, Expression patterns of identified trypsin genes in *P. tricornutum* based on qRT-PCR. The HNHP group was selected as control. Data are presented as mean values ± SD (n=3 biologically independent samples). The comparisons between the averages of the two groups were evaluated using the one-tailed Student's t test. The *P* values with significance ($p \leq 0.05$) are shown. Source data are provided as a Source Data file.

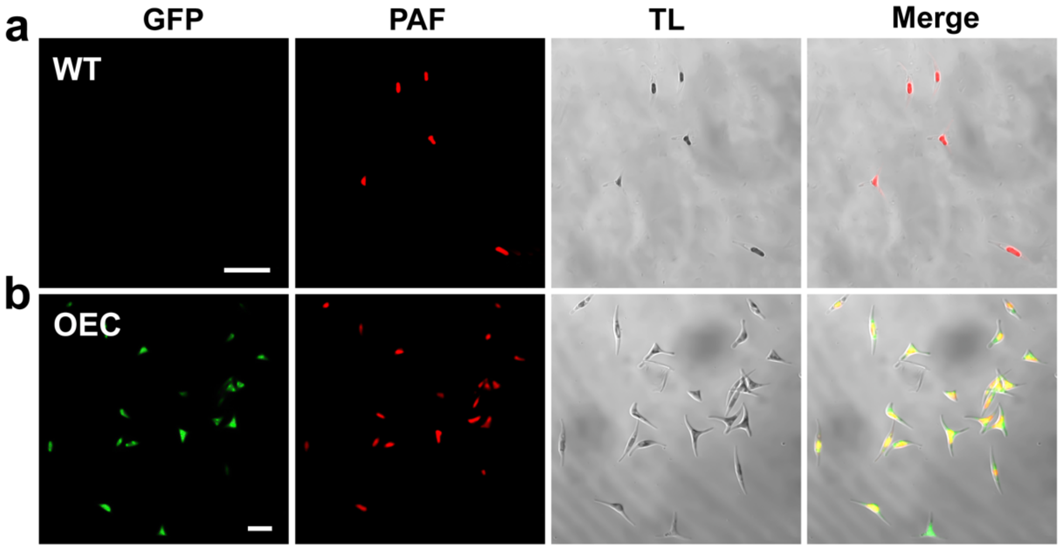

Supplementary Figure 6 Fluorescent microscopy of *P. tricornutum* cells. a, wild-type cells (WT) without any transformation. Scale bar, 10 µm. TL, transmission light. All experiments were repeated three times, and similar results were obtained. **b,** *P. tricornutum* WT cells transformed with pPha-T1-eGFP vector (as control of overexpression experiment, abbreviated as OEC). Scale bar, 10 µm. TL, transmission light. All experiments were repeated independently three times, and similar results were obtained.

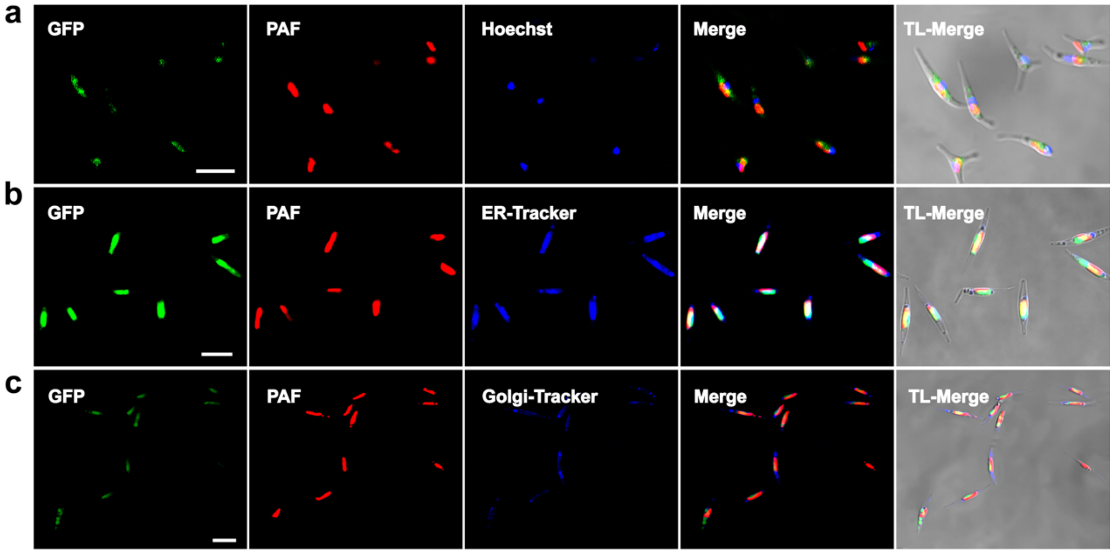

Supplementary Figure 7 Confocal image of the *PtTryp2*-OE cell line stained with different subcellular makers. a, Micrographs showing that the position of *PtTryp2* in the *PtTryp2-eGFP* transgenic cells (OE) does not overlap with the nuclear Hoechst 33342 staining but does partially overlap with plastid. All experiments were repeated independently three times, and similar results were obtained. **b**, The ER-Tracker Blue-White DPX showed the co-localization of ER and *PtTryp2*. All experiments were repeated independently three times, and similar results were obtained. **c**, The Golgi-Tracker staining in *PtTryp2-eGFP* transgenic cells reveals the localization of *PtTryp2* not in the Golgi. All experiments were repeated independently three times, and similar results were obtained. All images are representatives of multiple observations in several independent cultures. The *PtTryp2* signal is green (eGFP tag). ER, endoplasmic reticulum; GFP (green), green fluorescent protein; PAF (red), plastid autofluorescence; Scale bar, 10 μm. TL, transmission light. All experiments were repeated independently three times, and similar results were obtained.

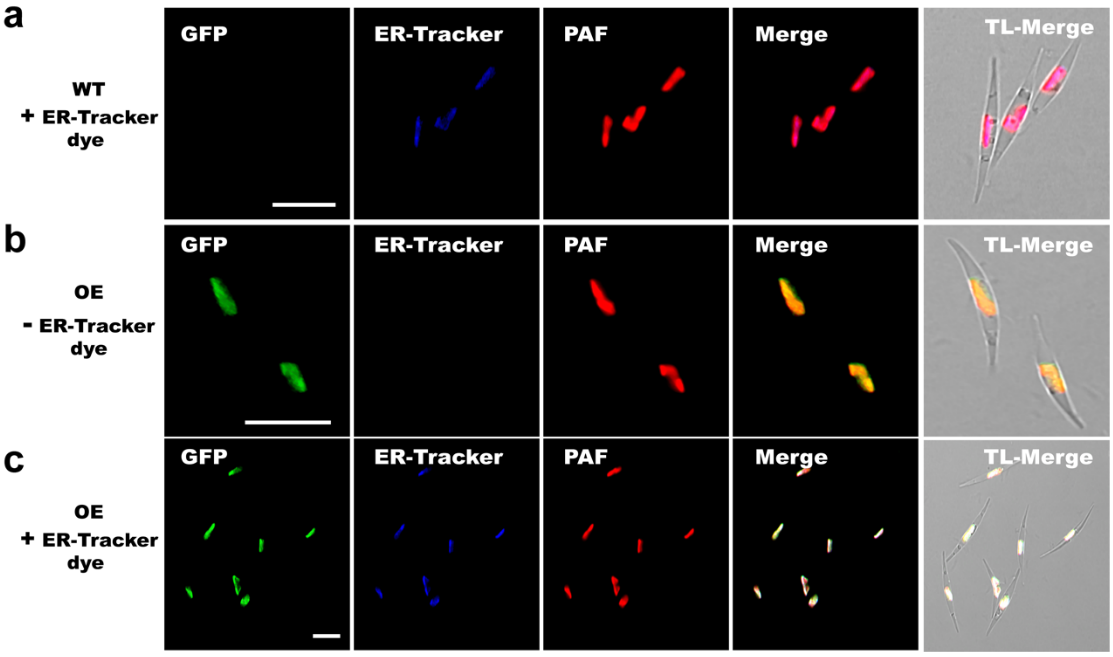

Supplementary Figure 8 Negative and positive controls for ER tracker. a, WT cell line stained with ER-Tracker Blue-White DPX. All experiments were repeated independently three times, and similar results were obtained. **b**, OE cell line without stained with ER-Tracker Blue-White DPX. All experiments were repeated independently three times, and similar results were obtained. **c**, OE cell line stained with ER-Tracker Blue-White DPX. All experiments were repeated independently three times, and similar results were obtained. All images are representatives of multiple observations in several independent cultures. ER, endoplasmic reticulum; GFP (green), green fluorescent protein; PAF (red), plastid autofluorescence; Scale bar, 10 µm. TL, transmission light.

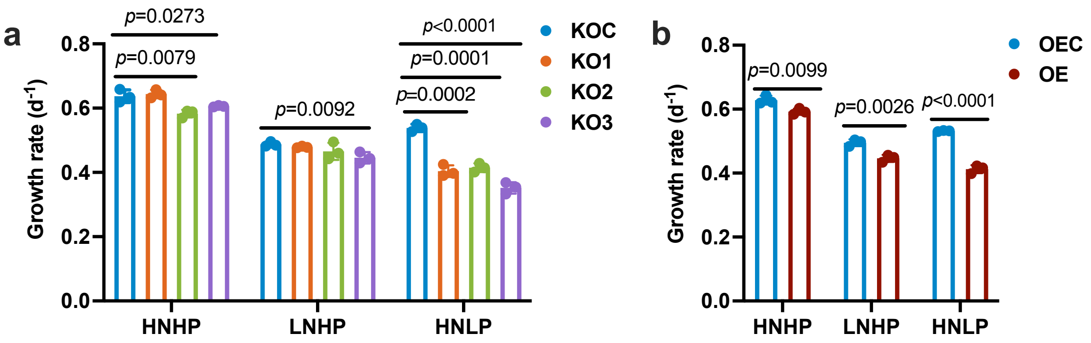

Supplementary Figure 9 The exponential growth rates (d1-d4) of different *PtTryp2* mutants under different N and P conditions. Nutrient conditions are indicated by HNHP (Nutrient-replete), LNHP (N-depleted, P-replete), HNLP (N-replete, P-depleted), and LNLP (Nutrient-depleted). **a**, The *PtTryp2*-knockout lines and KOC line. The KOC group was selected as control. Data are presented as mean values ± SD (n=3 biologically independent samples). The comparisons between the averages of the two groups were evaluated using the one-tailed Student's t test. The *P* values with significance ($p \leq 0.05$) are shown. **b**, The *PtTryp2*-overexpression line and OEC line. The OEC group was selected as control. Data are presented as mean values ± SD (n=3 biologically independent samples). The comparisons between the averages of the two groups were evaluated using the one-tailed Student's t test. The *P* values with significance ($p \leq 0.05$) are shown. Source data are provided as a Source Data file.

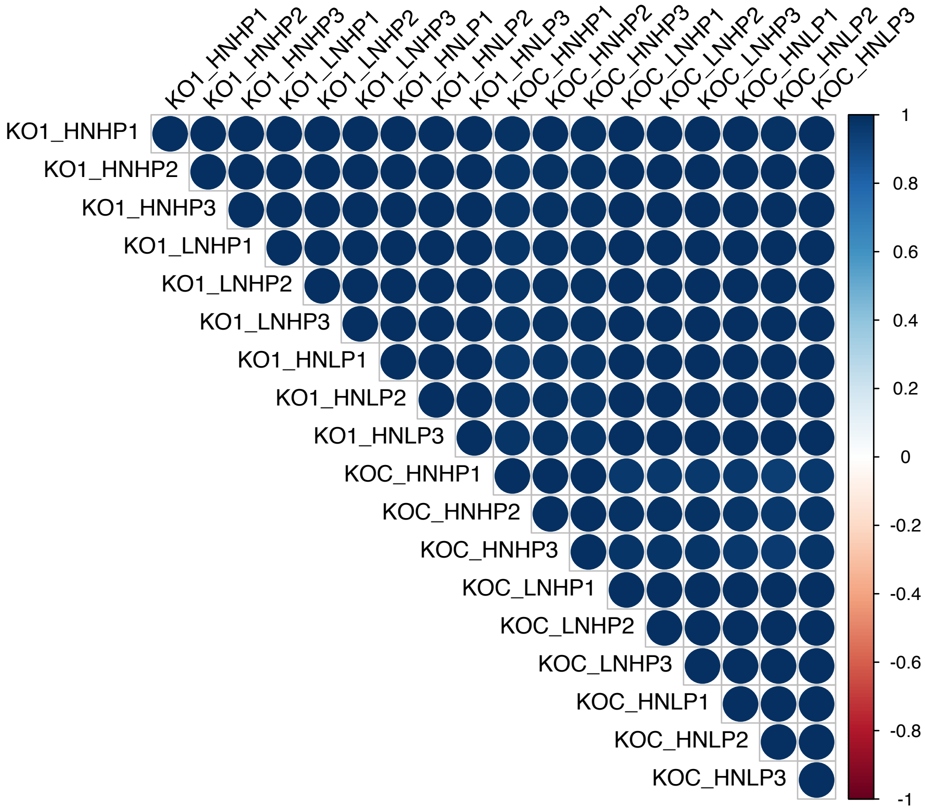

Supplementary Figure 10 Repeatability analysis for biological replicates between RNA-seq samples based on 11 classic housekeeping gene. The correlation analysis based on the selected housekeeping gene transcripts was very high (> 0.94), indicating that there is good reproducibility between samples. The 11 putative housekeeping gene lists were retrieved according to Siaut et al's research, detail gene information was listed in Supplementary Table 4. Source data are provided as a Source Data file.

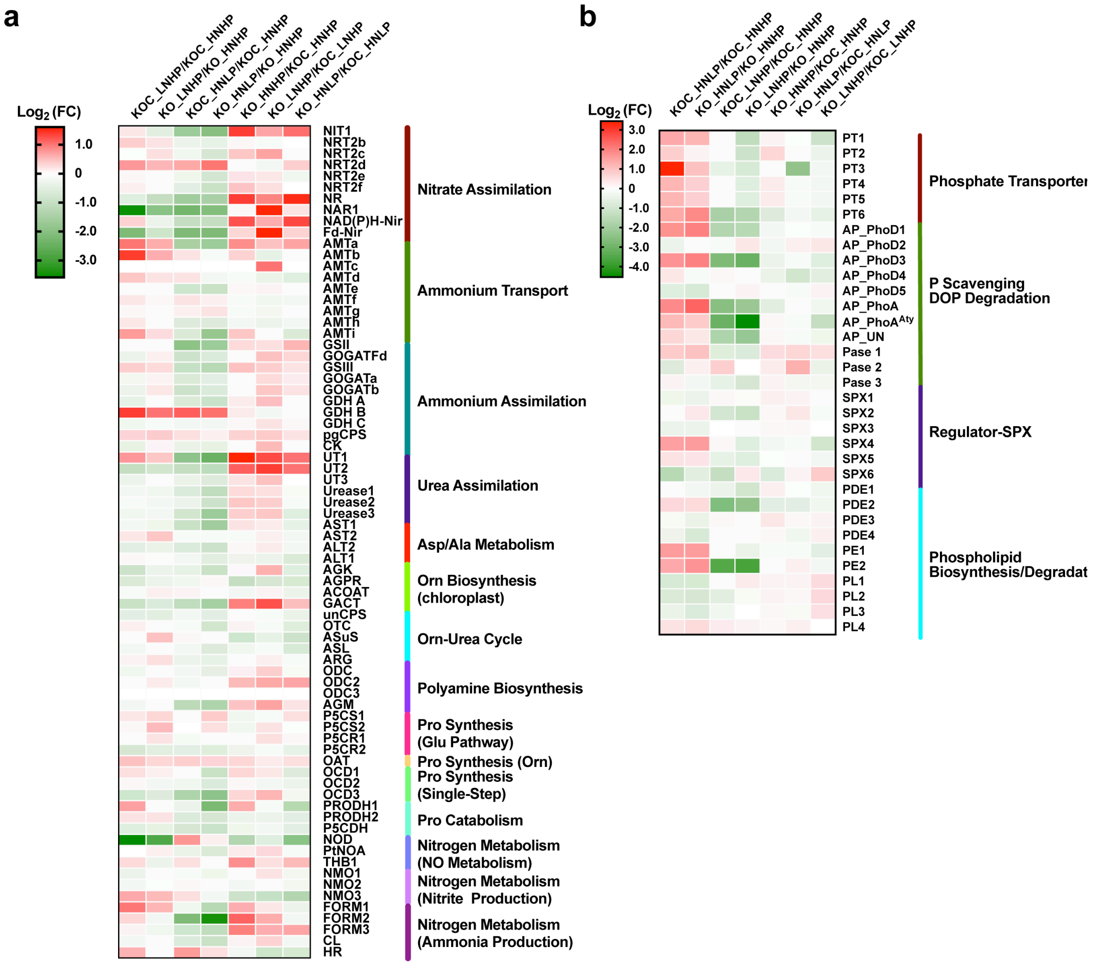

Supplementary Figure 11 Transcriptomic evidence that *PtTryp2* is involved in regulating nitrogen and phosphorus signaling and mediated crosstalk between N and P. a, Transcription of nitrogen assimilation genes and intersecting pathways. **b**, Transcription of phosphorus responsive genes. Heatmap shows log $_2$ (Fold change) for nitrogen and phosphorus responsive catalog genes. Nitrogen responsive catalog genes were selected as in Smith et al. (2019). Phosphorus responsive catalog genes were selected as in Sharma et al. (2020) and Zhang et al. (2021). Gene name abbreviations and gene id can be found in source data. Source data are provided as a Source Data file.

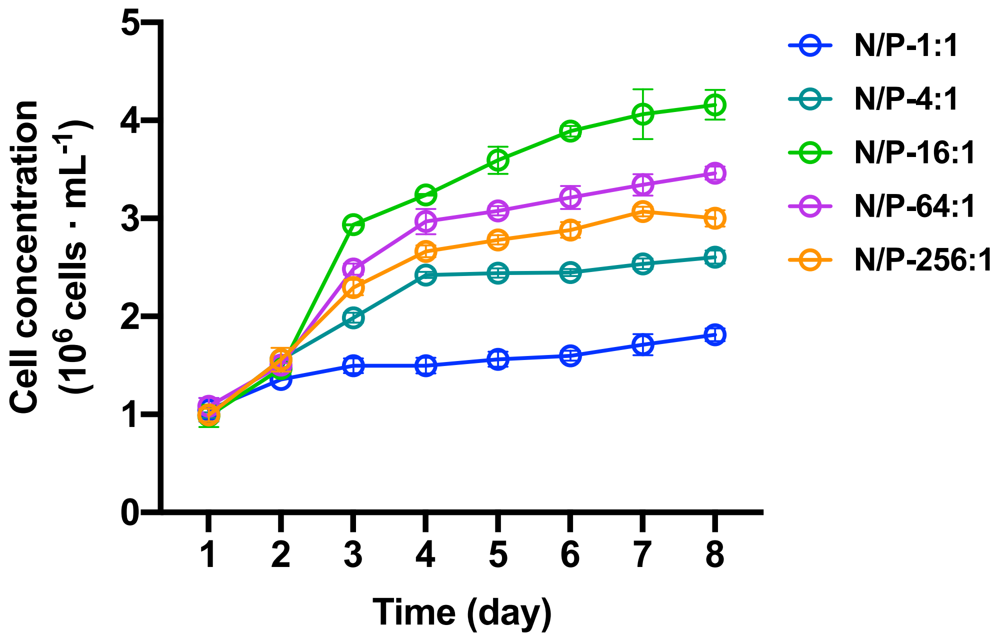

Supplementary Figure 12 Growth curves of wild type *P. tricornutum* across different N/P nutrient stoichiometric ratio conditions. The N/P nutrient ratio of 16:1 was optimal for *P. tricornutum* growth. The medium was the standard f/2 medium with different NO_3^- and PO_4^{3-} supply. N/P-1:1, 2.5 μM NO_3^- and 2.5 μM PO_4^{3-}. N/P-4:1, 10 μM NO_3^- and 2.5 μM PO_4^{3-}. N/P-16:1, 40 μM NO_3^- and 2.5 μM PO_4^{3-}. N/P-64:1, 40 μM NO_3^- and 0.625 μM PO_4^{3-}. N/P-256:1, 40 μM NO_3^- and 0.156 μM PO_4^{3-}. In order to show clear of the cell growth limited by nutrient depletion, all the y axis was set on the same scale. Data are presented as mean values ± SD (n=3 biologically independent samples). Source data are provided as a Source Data file.

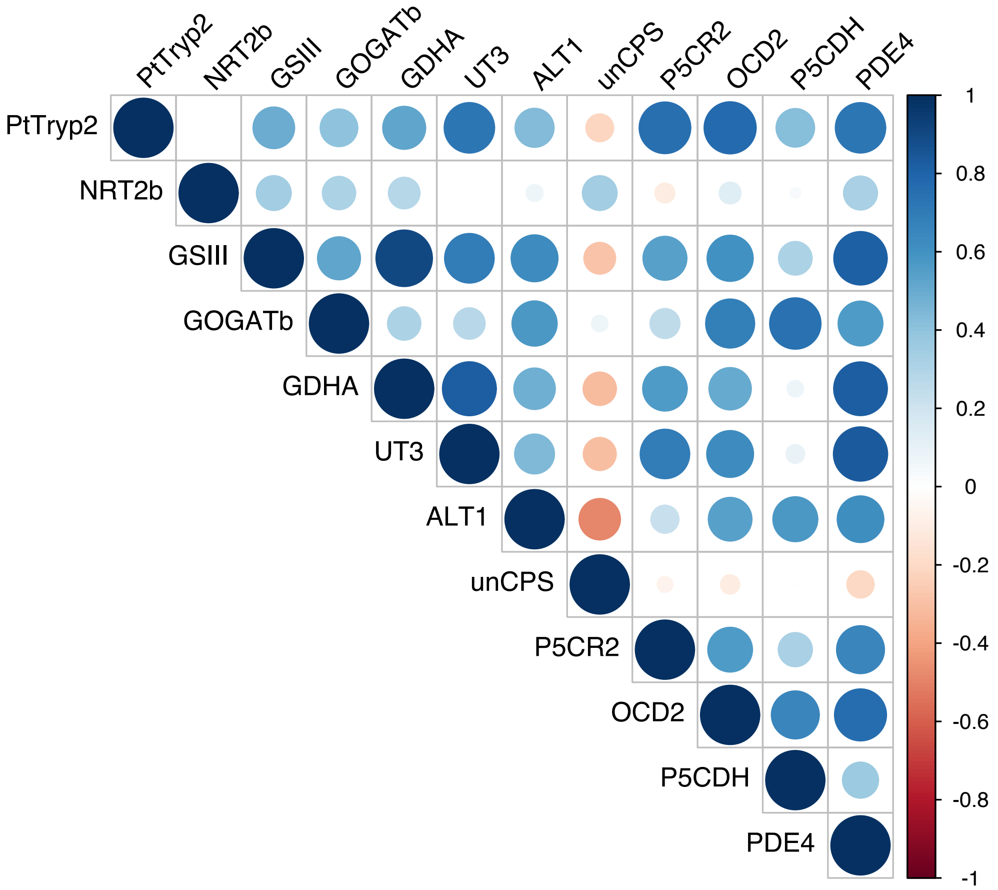

Supplementary Figure 13 The correlation coefficient of the expression of *PtTryp2* with that of N and P responsive genes. The *PtTryp2* expression levels show correlated best with several N and P responsive genes (UT3, P5CR2, OCD2 and PDE4). The N and P responsive genes were identified from the co-expression gene catalog co-expressed with *PtTryp2*. Gene name abbreviations and gene id can be found in Supplementary Table 2 and 3. Source data are provided as a Source Data file.

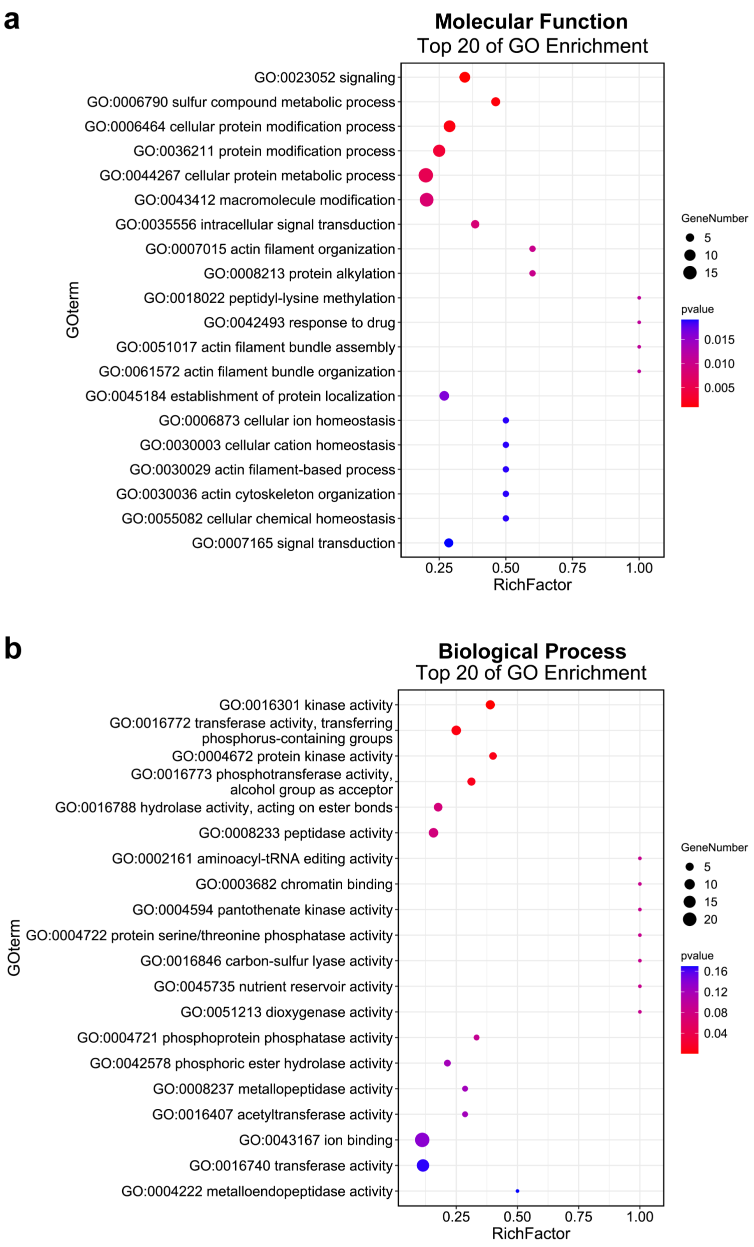

Supplementary Figure 14 Dot plot of the top 20 GO terms enriched by the *PtTryp2* co-expression gens. a, the molecular function subclasses of the enriched GO terms. The significance of enriched GO functional groups was evaluated using the Fisher's exact test with an adjusted P value ≤ 0.05. The size of the dots represents the number of genes and the color of the dots represent the adjusted P values. **b**, the biological process subclasses of the enriched GO terms. The functional enrichment of the gene set shows that *PtTryp2* possibly regulates post-transcriptional regulation pathway, intracellular signal transduction pathway and a set of kinases related to phosphorus metabolism and recycle pathway. The significance of enriched GO functional groups

was evaluated using the Fisher's exact test with an adjusted *P* value $\leq$ 0.05. The size of the dots represents the number of genes and the color of the dots represent the adjusted *P* values. The first four GO terms are closely related to cellular P recycle and metabolism.
