# Supplementary material for: Trypsin is a coordinate regulator of N and P nutrients in marine phytoplankton
Source: Nat Commun. 2022 Jul 12;13:4022. doi: 10.1038/s41467-022-31802-6 (PMC9276738; doi:10.1038/s41467-022-31802-6)
Supplement: Supplementary file 3 — Reporting Summary [file 41467_2022_31802_MOESM3_ESM.pdf]

nature portfolio

Corresponding author(s): Senjie Lin

Last updated by author(s): Jun 12, 2022

Reporting Summary

Nature Portfolio wishes to improve the reproducibility of the work that we publish. This form provides structure for consistency and transparency in reporting. For further information on Nature Portfolio policies, see our Editorial Policies and the Editorial Policy Checklist.

Statistics

For all statistical analyses, confirm that the following items are present in the figure legend, table legend, main text, or Methods section.

n/a	Confirmed	
☐	☒	The exact sample size (*n*) for each experimental group/condition, given as a discrete number and unit of measurement
☐	☒	A statement on whether measurements were taken from distinct samples or whether the same sample was measured repeatedly
☐	☒	The statistical test(s) used AND whether they are one- or two-sided<br>*Only common tests should be described solely by name; describe more complex techniques in the Methods section.*
☒	☐	A description of all covariates tested
☐	☒	A description of any assumptions or corrections, such as tests of normality and adjustment for multiple comparisons
☐	☒	A full description of the statistical parameters including central tendency (e.g. means) or other basic estimates (e.g. regression coefficient) AND variation (e.g. standard deviation) or associated estimates of uncertainty (e.g. confidence intervals)
☐	☒	For null hypothesis testing, the test statistic (e.g. *F*, *t*, *r*) with confidence intervals, effect sizes, degrees of freedom and *P* value noted<br>*Give P values as exact values whenever suitable.*
☒	☐	For Bayesian analysis, information on the choice of priors and Markov chain Monte Carlo settings
☒	☐	For hierarchical and complex designs, identification of the appropriate level for tests and full reporting of outcomes
☐	☒	Estimates of effect sizes (e.g. Cohen's *d*, Pearson's *r*), indicating how they were calculated

Our web collection on statistics for biologists contains articles on many of the points above.

Software and code

Policy information about availability of computer code

Data collection	Confocal images were collected and analyzed using Zen software (ZENblue3-1_ZENblack_3-0SR-lite). RNA-seq reads were aligned to the Phaeodactylum tricornutum (ASM15095v2) genome released in Ensembl (http://protists.ensembl.org/Phaeodactylum_tricornutum/Info/Index). Genes were identified by hmmer (Version 3.1) and confirmed by NCBI Conserved Domain Search (https://www.ncbi.nlm.nih.gov/Structure/cdd/wrpsb.cgi). Primers were designed by Primer Premier 5. Publicly-available worldwide distributions and abundances of trypsin genes were acquired from the OGA datasets (https://tara-oceans.mio.osupytheas.fr/ocean-gene-atlas/).
Data analysis	Data analyses were performed using Prism GraphPad (Version 9). The sequencing data was filtered with SOAPnuke (v1.5.2). Differentially expressed gene analysis was performed using DESeq2(v1.4.5). The expression level of a gene was calculated using RSEM (v1.2.12). Subcellular localization was predicted using NUCPRED, SIGNALP 3.0, SIGNALP 4.1, TMHMM v.2.0, TARGETP, CHLOROP, PROTEOME ANALYST, EUK-MPLOC, HECTAR v.1.3, and ASAFIND v.1.1.5. The web-based comprehensive tool RefFinder (http://blooge.cn/RefFinder/), which integrates currently available major computational program analysis (geNorm, Normfinder, BestKeeper, and the comparative Delta-Ct method) was used to assess the reference gene stability. The geographic distribution of trypsin in phytoplankton based on Tara Oceans datasets were visualized using the maps (version 3.4.0) R software package by RStudio (version 1.4.1717). Mantel correlations between trypsin mRNA abundance and environmental data using the vegan (version 2.5-7) R software package by RStudio (version 1.4.1717).

For manuscripts utilizing custom algorithms or software that are central to the research but not yet described in published literature, software must be made available to editors and reviewers. We strongly encourage code deposition in a community repository (e.g. GitHub). See the Nature Portfolio guidelines for submitting code & software for further information.

Data

Policy information about availability of data

All manuscripts must include a data availability statement. This statement should provide the following information, where applicable:

- Accession codes, unique identifiers, or web links for publicly available datasets
- A description of any restrictions on data availability
- For clinical datasets or third party data, please ensure that the statement adheres to our policy

The data, including the source data that supports the finding of this study, are available within the paper, its supplementary information files, or publicly available datasets. Publicly-available trypsin sequences were obtained from Pfam (https://pfam.xfam.org/family/PF00089 and https://pfam.xfam.org/family/PF13365), and Ensemble databases (http://protists.ensembl.org/Phaeodactylum_tricornutum/Info/Index). Publicly-available worldwide distributions and abundances of trypsin genes were acquired from the OGA datasets (https://tara-oceans.mio.osupytheas.fr/ocean-gene-atlas/).

Human research participants

Policy information about studies involving human research participants and Sex and Gender in Research.

Reporting on sex and gender	*Use the terms sex (biological attribute) and gender (shaped by social and cultural circumstances) carefully in order to avoid confusing both terms. Indicate if findings apply to only one sex or gender; describe whether sex and gender were considered in study design whether sex and/or gender was determined based on self-reporting or assigned and methods used. Provide in the source data disaggregated sex and gender data where this information has been collected, and consent has been obtained for sharing of individual-level data; provide overall numbers in this Reporting Summary. Please state if this information has not been collected. Report sex- and gender-based analyses where performed, justify reasons for lack of sex- and gender-based analysis.*
Population characteristics	*Describe the covariate-relevant population characteristics of the human research participants (e.g. age, genotypic information, past and current diagnosis and treatment categories). If you filled out the behavioural & social sciences study design questions and have nothing to add here, write "See above."*
Recruitment	*Describe how participants were recruited. Outline any potential self-selection bias or other biases that may be present and how these are likely to impact results.*
Ethics oversight	*Identify the organization(s) that approved the study protocol.*

Note that full information on the approval of the study protocol must also be provided in the manuscript.

Field-specific reporting

Please select the one below that is the best fit for your research. If you are not sure, read the appropriate sections before making your selection.

☐ Life sciences ☐ Behavioural & social sciences ☒ Ecological, evolutionary & environmental sciences

For a reference copy of the document with all sections, see nature.com/documents/nr-reporting-summary-flat.pdf

Ecological, evolutionary & environmental sciences study design

All studies must disclose on these points even when the disclosure is negative.

Study description	We find that the ancient enzyme trypsin occurs widely in global ocean phytoplankton. By CRISPR/Cas9 mediated-knockout and overexpression analyses coupled with physiological measurements, we show that trypsin is a coordinate regulator of nitrogen and phosphorus stoichiometric homeostasis in the diatom examined.
Research sample	This study involved only laboratory cultures, no environmental samples. The wild-type culture of Phaeodactylum tricornutum Bohlin strain was provided by the Center for Collections of Marine Algae, Xiamen University, China. The knockout strains (PtTryp2-KO) and PtTryp2-overexpression strain (PtTryp2-OE) were generated in this study using CRISPR/Cas9 gene editing technique with biolistic bombardment. All these cultures were grown under nutrient-replete, N-depleted, and P-depleted conditions to represent nutrient-sufficient and nutrient-limited conditions, each in triplicate, and samples were collected from each culture at the exponential (nutrient-replete cultures) or equivalent (N- and P- depleted cultures) stage for analyses.
Sampling strategy	After the cultures had been acclimated to the respective nutrient conditions (showing respective phenotypes), samples were collected for physiological measurements and molecular analyses. One triplicate set of samples from triplicate independent cultures were filtered onto the 3.0 μm polycarbonate membrane filters (Millipore) and resuspended in 1 mL Trizol Reagent (Invitrogen, Carlsbad, CA, USA), immediately frozen in liquid nitrogen and subsequently stored at -80°C for RNA isolation. The second triplicate set of samples were filtered onto pre-combusted 25 mm GF/F filters and subsequently stored at -20°C for cellular N and P content measurements. The third triplicate set of samples were filtered through 25 mm GF/F filter and 0.22 μm sterile filter, then stored at -20°C for NO3- and PO4+ concentration determination. For N and P content measurements, the minimal sample size was 1×10^7

	cells in each sample, and for RNA analyses, the minimal cell number for each sample was 1×10^8; these samples sizes are consistent with previous studies (Zhang et al., 2021).
Data collection	The datasets used for analyzing global ocean distribution of trypsin genes were obtained from the public databases generated by the TARA Oceans expedition team: the Marine Atlas of Tara Oceans Unigenes and eukaryotes metatranscriptomes (MATOUv1+metaT) (https://tara-oceans.mio.osupytheas.fr/ocean-gene-atlas/) and PhyloDB databases. To generate our own transcriptomic data, mRNA was purified using the Oligo(dT)-attached magnetic beads. The total RNA and isolated mRNA quality and quantity were checked using the Agilent 2100 Bioanalyzer and NanoDrop (Thermo Fisher Scientific, MA, USA). Libraries for RNAseq were created from 1μg of mRNA from each culture. The resulting libraries were loaded into the patterned nanoarray, and single-end 50 bp reads were generated on BGIseq500 platform (BGI-Shenzhen, China), with a data output of about 22 M total clean reads for each library. The clean reads were aligned to the Phaeodactylum tricornutum (ASM15095v2) genome released in Ensembl, done by BGI-Shenzhen, China.
Timing and spatial scale	This study is not a field study; therefore, timing and spatial scale is not relevant.
Data exclusions	No data were excluded in the entire study.
Reproducibility	All physiological parameters were measured in at least two experiments, each with three independent biological replicates. The results are all consistent. Transcriptome profiling was conducted in only one experiment; however, each culture condition had three independent biological replicates and they produced very similar results.
Randomization	All culture bottles were randomly placed in the incubator. All replicate cultures were sampled, with no selection involved. For each culture, samples were taken after the culture was gently and thoroughly mixed to make sure they represent the entire culture.
Blinding	Samples for measurements and analyses were blinded so that no information about the culture condition was indicated on the samples during the measurements and analyses.

Did the study involve field work? ☐ Yes ☒ No

Reporting for specific materials, systems and methods

We require information from authors about some types of materials, experimental systems and methods used in many studies. Here, indicate whether each material, system or method listed is relevant to your study. If you are not sure if a list item applies to your research, read the appropriate section before selecting a response.

Materials & experimental systems

n/a	Involved in the study
☐	☒ Antibodies
☒	☐ Eukaryotic cell lines
☒	☐ Palaeontology and archaeology
☒	☐ Animals and other organisms
☒	☐ Clinical data
☒	☐ Dual use research of concern

Methods

n/a	Involved in the study
☒	☐ ChIP-seq
☒	☐ Flow cytometry
☒	☐ MRI-based neuroimaging

Antibodies

Antibodies used	Anti-EGFP antibody and anti-GAPDH antibody used in this study were both commercialized antibody and were purchased from Abcam (England). Anti-EGFP body: Mouse monoclonal [F56-6A1.2.3] against EGFP, Abcam, Cat no. ab184601, Lot no.GR3277308-1, used in 1:1000 dilution. Anti-GAPDH antibody: Mouse monoclonal [FF26A] against GAPDH, Abcam, Cat no. ab59164, used in 1:10,000 dilution.
Validation	The antibodies for EGFP (Cat no. ab184601) and GAPDH (Cat no. ab59164) were purchased from Abcam (England). Please refer to the following links for the validation of the antibodies:<br>Anti-EGFP antibody: https://www.abcam.com/egfp-antibody-f56-6a123-ab184601.html<br>Anti-GAPDH antibody: https://www.abcam.com/gapdh-antibody-ff26a-ab59164.html
